# LICC: L-BLP25 in patients with colorectal carcinoma after curative resection of hepatic metastases--a randomized, placebo-controlled, multicenter, multinational, double-blinded phase II trial

**DOI:** 10.1186/1471-2407-12-144

**Published:** 2012-04-11

**Authors:** Carl Christoph Schimanski, Markus Möhler, Michael Schön, Eric van Cutsem, Richard Greil, Wolf Otto Bechstein, Susanna Hegewisch-Becker, Götz von Wichert, Matthias Vöhringer, Michael Heike, Volker Heinemann, Marc Peeters, Stephan Kanzler, Stefan Kasper, Friedrich Overkamp, Jan Schröder, Daniel Seehofer, Frank Kullmann, Bernhard Linz, Irene Schmidtmann, Victoria Smith-Machnow, Ines Gockel, Hauke Lang, Peter R Galle

**Affiliations:** 1 First Deptartment of Internal Medicine, University Medical Center (UMC), University Hospital of Mainz, Mainz, Germany; 2 Department of General and Abdominal Surgery, Municipal Hospital of Karlsruhe, Karlsruhe, Germany; 3 Digestive Oncology Unit, University Hospital Gasthuisberg, Leuven, Belgium; 4 Third Department of Internal Medicine, University Medical Center (UMC), University Hospital of Salzburg, Salzburg, Austria; 5 Department of General and Abdominal Surgery, University Medical Center (UMC), University Hospital of Frankfurt, Frankfurt, Germany; 6 Onkologische Schwerpunktpraxis Eppendorf, Hamburg, Germany; 7 First Department of Internal Medicine, University Medical Center (UMC), University Hospital of Ulm, Ulm, Germany; 8 Center of Internal Medicine, Robert-Bosch Hospital, Stuttgart, Germany; 9 Med. Klinik Mitte, Hospital of Dortmund, Dortmund, Germany; 10 Third Department of Internal Medicine, University Hospital of Großhadern, Munich, Germany; 11 Department of Oncology, Antwerp University Hospital, Edegem, Belgium; 12 Department of Internal Medicine, Leopoldina Hospital, Schweinfurt, Germany; 13 Department of Internal Medicine, University Medical Center (UMC), University Hospital of Essen, Essen, Germany; 14 Oncologianova GmbH, Recklinghausen, Germany; 15 Outpatient center for hematology and oncology, Mühlheim an der Ruhr, Germany; 16 Department of General, Visceral and Transplantation Surgery, Campus Virchow Klinikum, Humboldt University, Berlin, Germany; 17 First Department of Internal Medicine, Kliniken Nordoberpfalz AG, Klinikum Weiden, Germany; 18 Outpatient center for hematology and oncology, Offenburg, Germany; 19 Institute of Medical Biometrics, Epidemiology and Informatics (IMBEI), University Medical Center (UMC), University Hospital of Mainz, Mainz, Germany; 20 iOMEDICO, Freiburg, Germany; 21 Department of General and Abdominal Surgery, University Medical Center (UMC), University Hospital of Mainz, Mainz, Germany; 22 First Dept. of Internal Medicine, University Medical Center (UMC), University Hospital of Mainz, Langenbeckstrasse 1, D-55131 Mainz, Germany

## Abstract

**Background:**

15-20% of all patients initially diagnosed with colorectal cancer develop metastatic disease and surgical resection remains the only potentially curative treatment available. Current 5-year survival following R0-resection of liver metastases is 28-39%, but recurrence eventually occurs in up to 70%. To date, adjuvant chemotherapy has not improved clinical outcomes significantly. The primary objective of the ongoing LICC trial (**L**-BLP25 **I**n **C**olorectal **C**ancer) is to determine whether L-BLP25, an active cancer immunotherapy, extends recurrence-free survival (RFS) time over placebo in colorectal cancer patients following R0/R1 resection of hepatic metastases. L-BLP25 targets MUC1 glycoprotein, which is highly expressed in hepatic metastases from colorectal cancer. In a phase IIB trial, L-BLP25 has shown acceptable tolerability and a trend towards longer survival in patients with stage IIIB locoregional NSCLC.

**Methods/Design:**

This is a multinational, phase II, multicenter, randomized, double-blind, placebo-controlled trial with a sample size of 159 patients from 20 centers in 3 countries. Patients with stage IV colorectal adenocarcinoma limited to liver metastases are included. Following curative-intent complete resection of the primary tumor and of all synchronous/metachronous metastases, eligible patients are randomized 2:1 to receive either L-BLP25 or placebo. Those allocated to L-BLP25 receive a single dose of 300 mg/m^2 ^cyclophosphamide (CP) 3 days before first L-BLP25 dose, then primary treatment with s.c. L-BLP25 930 μg once weekly for 8 weeks, followed by s.c. L-BLP25 930 μg maintenance doses at 6-week (years 1&2) and 12-week (year 3) intervals unless recurrence occurs. In the control arm, CP is replaced by saline solution and L-BLP25 by placebo. Primary endpoint is the comparison of recurrence-free survival (RFS) time between groups. Secondary endpoints are overall survival (OS) time, safety, tolerability, RFS/OS in MUC-1 positive cancers. Exploratory immune response analyses are planned. The primary endpoint will be assessed in Q3 2016. Follow-up will end Q3 2017. Interim analyses are not planned.

**Discussion:**

The design and implementation of such a vaccination study in colorectal cancer is feasible. The study will provide recurrence-free and overall survival rates of groups in an unbiased fashion.

**Trial Registration:**

EudraCT Number 2011-000218-20

## Background

The primary objective of the ongoing LICC trial (**L**-BLP25 **I**n **C**olorectal **C**ancer) is to determine whether L-BLP25, an active cancer immunotherapy, extends recurrence-free survival (RFS) time over placebo in colorectal cancer pts following R0/R1 resection of hepatic metastases. Stimuvax^® ^(BLP25 Liposome Vaccine or L-BLP25) is an investigational therapeutic cancer vaccine co-developed by Oncothyreon Canada Inc. (formerly Biomira Inc., Edmonton, Canada), and Merck KGaA, Darmstadt, Germany for the use as an active specific immunotherapy for MUC1- expressing tumors. Details of the physical, chemical and pharmaceutical properties, the non-clinical trials and effects and safety in humans have been published elsewhere 2008) [1].

Colorectal cancer is amongst the three most frequent malignancies in Western countries [2,3]. Survival is delineated by local recurrence, by lymphatic and predominantly by hematogenous dissemination [4]. Mutations in tumor suppressor genes (APC, DCC, Smad-2, Smad-4, p53) and oncogenes (K-ras) are molecular determinants occurring during the development of sporadic colorectal cancer, which was first summarized in the adenoma-carcinoma sequence described by Vogelstein et al. [5-7]. Since only 8% of colorectal cancers harbor concomitant mutations of APC, K-ras, and p53, it seems very likely that additional pathogenic alterations instrumentally mediate progression and metastasis of colorectal cancer [8].

At the time of first diagnosis, about 35% of colorectal cancer patients have distant metastases [9]. Distant metastases limited to the liver occur in 15-20% of all patients initially diagnosed with colorectal cancer [10-12]. However, only 15-20% of synchronic hepatic metastases are resected by surgery. Complete surgical resection of hepatic metastases represents the only curative option: cure is otherwise not attainable. The 5-year survival after R0-resection of liver metastases ranges between 28% to 39% [13,14]. Unfortunately, recurrence rates after R0 resection of hepatic metastases peak at up to 70% in the long-term follow-up [15].

The decision as to whether metastases are to be treated by surgical resection or by other therapeutic options such as neoadjuvant chemotherapy, should be discussed and determined by interdisciplinary tumor boards [9]. The requirements for surgical resections are (i) that there is no evidence of extra-hepatic tumor, (ii) less than 70% of the liver is tumor-bearing, (iii) fewer than 3 liver veins and fewer than 7 segments of the liver are affected. Further exclusion criteria for surgical resection of liver metastases are the presence of CHILD B/C cirrhosis and/or other severe concomitant diseases [16]. Using scoring systems such as that described by Fong, the prognosis can be estimated preoperatively [17]. The existence of at least two Fong points predicts a median survival of 47 months after surgical resection of liver metastasis. However, the Fong score is only an indirect correlation of evaluated parameters (N-stage, size and number of metastases, preoperative CEA value and duration of disease-free interval) with the probability of incidence of organ metastasis. Organ-bound micro-metastases (MM) or disseminated tumor cells (DTC) are considered precursors of metachronic solid liver metastases [18-20].

A relevant clinical problem after primary or secondary resection of hepatic metastases is the high recurrence rate, ranging up to 70% [15]. According to de Jong and colleagues, the median RFS after resection of liver metastases is 23 months andthe median OS 36 months [15]. The application of classical chemotherapeutic strategies has not been sufficiently successful. Adjuvant 5-FU has non-significantly increased recurrence free survival (RFS 1.6 yrs. vs. 2.3 yrs.; P = 0.06), however this did not translate into a prolonged overall survival (4.0 vs. 5.0 years; P = 0.09) [21]. Similar data was published by Portier et al., prolonging the 5-year-RFS (33.5% vs. 26.7%; P = 0.028) without augmenting the 5-year-survival (51.1% vs. 41.1% P = 0.13) by an adjuvant 5-FU therapy (EBM grade 2b) [22]. Parks et al. found that a 5-FU based adjuvant chemotherapy versus watch and wait significantly improved postoperative overall survival (P = 0.007); however these data were retrospectively analysed registered data and thus not applicable (EBM grade 3b) [23].

Nordlinger's approach using peri-operative FOLFOX non-significantly increased the 3-yrs RFS by 7.3% (28.1% vs. 35.4%;P = 0.058); however patients treated per protocol (per protocol analysis) benefited even more (9.2%; 3-yrs. RFS 33.2% vs. 42.2%; P = 0.025) (EBM grade 1b) [24]. Other studies are limited by small numbers and do not augment EBM grade 2b [25,26].

The German S3 Guideline recommends surgery of primary resectable liver metastases (recommendation grade A; evidence grade 3b; strong consensus). In contrast, a neoadjuvant chemotherapy of primary resectable metastases is considered optional and is limited to well-defined, exceptional cases (recommendation grade 0; evidence grade 3, strong consensus). Postoperative adjuvant chemotherapy after curative R0 resection of hepatic metastases is considered optional ("can be considered"; recommendation grade B; evidence grade 2, strong consensus) [9].

In contrast, according to the current consensus of the "European colorectal metastases treatment group" use of neoadjuvant/peri-operative combination chemotherapy (e.g. FOLFOX) is suggested in the case of primary resectable liver metastases whereas for potentially resectable liver metastases a neo-adjuvant combination chemotherapy (e.g. FOLFOX or FOLFIRI) plus application of a targeted therapy (bevacizumab, cetuximab, panitumumab) should be considered. Isolated adjuvant strategies are not recommended by the working group.

In summary, no generally accepted standard of care is available following curative-intent resection of hepatic metastases in colorectal cancer patients. L-BLP25 is a therapeutic vaccine that targets MUC1, a well known tumor-associated antigen. Recently, it has been shown that MUC1 is associated with cellular transformation as demonstrated by tumorigenicity [27] and can confer resistance to genotoxic agents [28]. High levels of MUC1 cell surface expression [29,30], reported immunosuppressive activities of its released ectodomain [31] and anti-adhesive properties [32,33] all contribute to the ability of the MUC1 antigen to protect and promote tumor cell growth and survival, making MUC1 an attractive target for cancer immunotherapy.

Based on these results, L-BLP25 has a promising potential as adjuvant therapy after curative-intent resection of hepatic metastases in colorectal cancer patients.

## Methods/Design

### Investigational medicinal product

**Figure 1:**
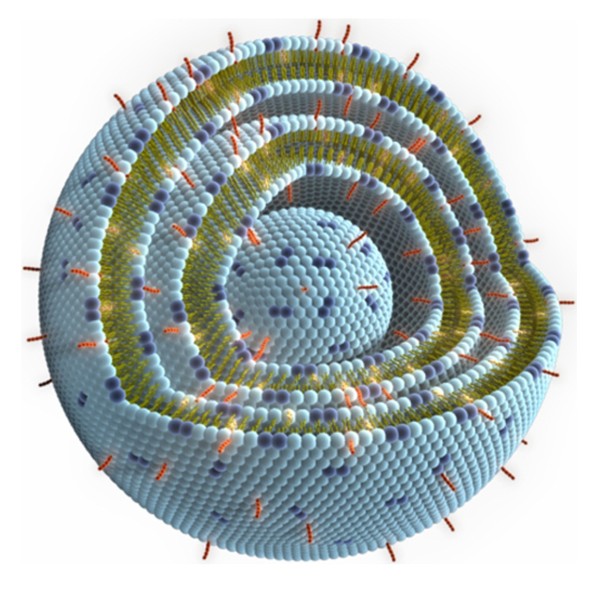
**L-BLP25**.

L-BLP25 is a lyophilized preparation, formulated to contain 241 μg of BLP25 lipopeptide and 128 μg of immunoadjuvant monophosphoryl lipid A in a 5 mL glass vial. The vial also contains 13,63 mg of liposomal lipids consisting of dipalmitoyl phosphatidylcholine (DPPC), cholesterol and dimyristoyl phosphatidylglycerol (DMPG). The BLP25 lipopeptide and monophosphoryl lipid A (MPL) are associated with the lipid bilayer of the liposomes that are formed upon rehydration of the dry powder (Figure 1). The active mechanism of L-BLP25 is mediated by the entire liposome formed after reconstitution. Only the entire liposomal formulation, in which the antigen and adjuvant are integrated into the lipid bi-layer, is capable of inducing the desired immune response against MUC1. L-BLP25 was designed principally to induce a cellular immune response to tumor tissues that express the MUC1 antigen. This cellular response is characterized by the proliferation of CD4-positive T cells in response to peptide, along with production of gamma interferon and the generation of cytotoxic T lymphocytes (CTLs) capable of killing MUC1-expressing tumor cells. The BLP25 lipopeptide provides the antigenic specificity for the T cell response, while the monophosphoryl lipid A serves as an adjuvant to enhance the cellular immune responses. The liposomal delivery system is designed to facilitate uptake of the vaccine by antigen-presenting cells (APCs) delivering the lipopeptide into the intracellular space, finally leading to presentation of peptides via class I and class II molecules of the HLA complex. This is expected to elicit a MUC1-specific cellular immune response mediated by T lymphocytes, including a CTL response. Further information on the immunological background supporting L-BLP25 and information on the immunization schedule can be found in the Investigators Brochure. Humoral responses to L-BLP25 are not expected, based on the specific formulation of the vaccine, and were found in only a few patients at low levels in previous clinical trials (EMR 63325-002, -003, and -004). Additional file 1: Table S1 provides an outline of the clinical trials conducted with L-BLP25 to date.

### Trial objectives

Primary objective is the comparative evaluation of recurrence-free survival (RFS) time between the treatment groups (L-BLP25 plus cyclophosphamide versus placebo vaccination and saline infusion). Secondary objectives are the comparative assessment of overall survival (OS) time, safety/tolerability, recurrence-free survival time in the subgroup of MUC1 positive cancers, and overall survival in the subgroup of MUC1 positive cancers. MUC1 expression analysis and immuno-monitoring parameters are to be defined in separate translational protocol.

### Trial design and plan

This is a randomized, placebo-controlled, multicenter, double-blinded, efficacy/safety study of L-BLP25 in patients with complete-resected metastatic CRC. Eligible patients will have had their primary tumor resected and have undergone curative-intent resection of liver metastases within the last 6 weeks. Eligible patients will be randomized to treatment with L-BLP25 vaccine versus placebo (2:1). Treatment will be discontinued upon documented relapse or, if subjects are free of relapse, treatment will be discontinued 3 years after the first vaccination of the maintenance phase. Randomization of 159 subjects (106 to the investigational arm and 53 to the control arm) was calculated. The enrollment period is expected to run from Q3 2011 to Q3 2013. The sample size considerations are based on the following assumptions:

• 2:1 randomization (106 pts. L-BLP25 vs. 53 pts. placebo)

• Increase in relapse-free time from 18 months under placebo to 24 months under L-BLP25 (i.e. a hazard ratio of 0.75)

• Linearly increasing (cumulative) enrollment rate over 24 months

• 60 months of total trial duration (i.e., a minimum follow-up of 36 months after the last patient included).

• α = 0.1 (1-sided) used for planning, which translates to a 2-sided α-level of 20%

• Power 59%

• HR 0.75

• Lost to follow-up hazard rate 0.0027 (6% drop out proportion at 60 months)

Follow-up will be event-driven and analysis is foreseen after approximately 113 events (recurrences) are reported unless this has still not occurred after completion of treatment of the last patient (i. e. 36 months after randomisation of the last active patient, which is approximately 60 months after initiation of the study). Based on a median RFS time of 18 months in the control arm and linearly increasing (cumulative) enrollment rate over 24 months, final analysis is projected at approximately 60 months post trial initiation. Assuming approximately 6% lost to follow-up, a total of 159 subjects (106 subjects in the L-BLP25 arm and 53 subjects in the control arm) will be enrolled to achieve the specified number of events in the scheduled follow-up time.

A Data Safety Monitoring Board (DSMB), monitoring the trial periodically, will ensure continued subject safety as well as the validity and scientific merit of the trial.

### Diagnosis and main inclusion and exclusion criteria

This trial is designed for patients with metastatic colorectal carcinoma (CRC), who have undergone a complete resection of their primary tumor and recent resection of their liver metastases (R0 or R1) with curative intent. Eligibility criteria for the LICC-trial are depicted in Table 1 and 2.

**Table 1:** Eligibility criteria for the LICC-trial

• Signed written informed consent.
• Male or female.

• At least 18 years of age.

• Female patients of childbearing potential (and if appropriate male patients with female partners of childbearing potential) must be willing to use an adequate method of contraception for 4 weeks prior to, during and 12 weeks after the last dose of trial medication. A negative pregnancy test is required for female subjects. Adequate contraception for female subjects is defined as two barrier methods, or one barrier method with a spermicide, or intrauterine device or use of hormonal female contraceptive. For the purpose of this trial, women of childbearing potential are defined as: "All female subjects after puberty unless they are post-menopausal for at least two years, are surgically sterile or are sexually inactive."

• Histologically confirmed diagnosis of adenocarcinoma of the colon or rectum with complete resection of primary tumor and no evidence of local relapse.

• Metastatic disease of the liver, with recent (< 6 weeks prior to randomization) resection (R0 or R1) of all liver metastases. Metastasectomy may have been either synchronous or metachronous. Any neoadjuvant therapy may have been applied for maximal 3 months prior to metastasectomy.

• Subject has had a colonoscopy or rectoscopy within the last three months prior to initiation of therapy

• Subject has an ECOG performance status of 0 or 1.

• Subject has adequate hematologic, hepatic, and renal function within 2 weeks prior to initiation of therapy as defined by the following:

- Absolute neutrophils > 1,500/mm^3 ^and platelets > 140,000/mm^3^. - Bilirubin < 1.5 × upper limit of normal (ULN). - AST and ALT < 2.5 × ULN. - Creatinine < 1.5 × ULN. - International Normalized Ratio (INR) and partial thromboplastin time (PTT) in the normal range of the local lab.

• Willingness to comply with study protocol requirements.

Inclusion criteria (Each of these to be met)

**Table 2:** Eligibility criteria for the LICC-trial

• Metastases other than liver metastases.
• R2 and Rx resected liver metastases. Patients with R1 resected liver metastases can be included if a further surgical resection is seen as not indicated or necessary in the surgeon's opinion.

• Chemotherapy within 4 weeks prior to randomization.

• Receipt of immunotherapy (e.g. interferons, tumor necrosis factor, interleukins, or growth factors [GM-CSF, G-CSF, M- CSF], monoclonal antibodies) within 4 weeks (28 days) prior to randomization.

• Any known autoimmune disease, past or current.

• A recognized immunodeficiency disease including cellular immuno-deficiencies, hypogammaglobulinemia or dysgammaglobulinemia; hereditary or congenital immunodeficiencies.

• Known or newly diagnosed active hepatitis B infection and/or hepatitis C infection, autoimmune hepatitis, known human immunodeficiency virus infection, or any other infectious process that in the opinion of the investigator could compromise the subject's ability to mount an immune response, or expose him/her to likelihood of more and/or severe side effects.

• Past or current history of malignant neoplasm other than CRC, except for curatively treated non-melanoma skin cancer, in-situ carcinoma of the cervix or other cancer curatively treated and with no evidence of disease for at least 5 years.

• Medical or psychiatric conditions that would interfere with ability to provide informed consent, communicate side effects, or comply with protocol requirements.

• Clinically significant cardiac disease, e.g. cardiac failure of New York Heart Association classes III-IV; uncontrolled angina pectoris, uncontrolled arrhythmia, uncontrolled hypertension, myocardial infarction in the previous 12 months as confirmed by an ECG.

• Splenectomy.

• Previous (less than 4 weeks prior to randomization) or concurrent treatment with a non-permitted drug.

• Pregnancy and lactation period.

• Participation in another clinical study within 30 days prior to randomization.

• Known hypersensitivity to the study treatment drugs.

• Known alcohol or drug abuse.

• Legal incapacity or limited legal capacity.

• Any other reason that, in the opinion of the investigator, precludes the patient from participating in this study.

Exclusion criteria

#### Pre-treatment evaluations

After an informed consent form has been signed by the subject, following evaluations will be undertaken within 28 days prior to the first administration of L-BLP25 or placebo, and prior to the administration of cyclophosphamide or saline solution: medical history, family history of autoimmune disease, vital signs, physical examination (including height, weight and a limited neurological examination), electrocardiogram (ECG), ECOG performance status, imaging (computerized tomography/magnetic resonance imaging (CT/MRI), baseline hepatic ultrasound, Fong score and pregnancy test. Within 14 days prior to the first administration of L-BLP25 or placebo and prior to administration of cyclophosphamide or placebo, blood sampling for hematology/serum biochemistry, coagulation, CEA, CA19-9, ANA, SMA, SLA, LKM, AMA, ANCA, Anti-HBc-IgG, Anti-HBc-IgM, Anti-HBs, HBs antigen and anti-HCV will be performed (total blood volume for Pre-treatment evaluations: 30 ml). A tumor sample (paraffin fixed) must be available for immunohistochemical MUC1 analysis. The MUC1 analysis will be carried out in the central laboratory at Sponsor's site, every other standard laboratory analysis will be done in the local laboratories.

#### Randomization

If the subject is deemed eligible, randomization will be performed using a 2:1 ratio (Investigational arm: Placebo arm) by the help of an IVRS.

#### Cyclophosphamide administration visit

Within 7 days of randomization and 3 days (72 hours ± 8 hours) prior to the first application of L-BLP25 or placebo, subjects will receive a single intravenous (I.V.) infusion of a 300 mg/m^2 ^(maximum 600 mg) cyclophosphamide (treatment arm) or a corresponding volume of saline solution (control arm).

#### Primary treatment phase

Subjects will receive a target total 8 subcutaneous vaccinations of L-BLP25 or placebo at weekly intervals (primary treatment phase). Each vaccination consists of 4 subcutaneous injections. Injection sites will be inspected 30 minutes after injection.

The 5th visit of the vaccination schedule will additionally include a physical examination, vital signs, ECOG performance status, CEA, CA19-9, ANA, hematology/serum biochemistry and coagulation.

A hepatic ultrasound will additionally be performed at visit 8.

Evaluation after Primary Treatment Phase will take place 1 week after the completion of the primary treatment phase (8^th ^vaccination). During this visit the following tasks will be performed: physical examination, vital signs, ECG, ECOG performance status, hematology/serum biochemistry, coagulation, CEA, CA19-9 and ANA (total 30 ml blood volume), injection site inspection.

#### Maintenance treatment phase

Subjects will receive vaccinations at 6-week intervals during year 1 and 2, commencing 6 weeks after the end of the primary treatment phase. Vaccination intervals will be 12 weeks in year 3. Subjects will receive vaccinations until documentation of recurrence or the patient discontinues (whatever is earlier). Treatment will end 3 years after the first vaccination of the maintenance treatment phase. Vital signs will be taken and injection sites will be inspected 30 minutes after vaccination. In addition, starting with the end of the primary treatment phase, a physical examination, ECOG performance status, CEA, CA19-9, ANA, hematology/serum biochemistry, coagulation (total 30 ml blood volume per assessment) and a serum or urine pregnancy test (women of childbearing potential only) will be performed every 12 weeks, plus additional sampling for hematology/biochemistry only every intermediate 6 weeks until after week 92 (total blood volume 12 ml per assessment). Vaccination intervals will be 6 weeks during years 1 and 2 after first vaccination and 12 weeks during year 3 after first vaccination.

#### End of treatment evaluation

When recurrence has been documented or the subject is discontinued, study treatment is to be discontinued. 12 weeks after the last treatment, an End of Treatment Evaluation will be performed, consisting of vital signs, physical examination, ECOG performance status, hematology/serum biochemistry, coagulation, ECG, CEA, CA19-9, ANA (total 30 ml blood volume), serum or urine pregnancy test (women of childbearing potential only), CT/MRI, hepatic ultrasound and injection site inspection. When second-line treatment is indicated, and initiation is planned less than 12 weeks after the last treatment, then the end of treatment evaluation will be performed prior to the intitation of second-line therapy.

#### Long term follow-up

After the last treatment, i.e. after documented relapse or after the subject or investigator has decided to stop treatment, the investigator or his/her designee will contact the subject every 6 months for documentation of recurrence status and survival status. The follow-up for all patients ends 4 years after the last active patient in the maintenance treatment phase has received the first dose of the maintenance therapy (i.e. had had two such follow-up assessments). All SAEs persisting at the end of the study will be followed up until resolved/stabilized or an alternative cause is found. At the first long term follow-up 6 months after last treatment general autoimmune phenomena will be queried.

#### Safety assessment and concomitant treatment

AE assessment and recording of concomitant medication and concurrent procedures will be done during all visits in both treatment phases. During long term follow-up, AEs which are considered to be related to the investigational product will be recorded.

#### Lab assessments and total blood volume collected

Standard laboratory assessments include serum biochemistry, hematology, coagulation, CEA, CA19-9 and ANA. Prior to first treatment additionally to the standard laboratory assessment ANA, SMA, SLA, LKM, AMA, ANCA, AntiHBc-IgG, Anti-HBc-IgM, anti-HBs, HBs antigen and anti-HCV are to determined. A planned maximum of 564 ml blood will be drawn during the clinical trial; in the case of AE monitoring additional blood samples may be drawn. Respectively, 30 ml per assessment will be drawn for the pre-treatment evaluation, at week 5 of the primary treatment phase, 1 week after the primary treatment phase, at 12 different time points during the maintenance treatment phase (weeks 14, 26, 38, 50, 62, 74, 86, 98, 110, 122, 134 and 146) and at the end of treatment evaluation. During the maintenance phase, additional 12 ml sampling per assessment for biochemistry and hematology only will be drawn at weeks 20, 32, 44, 56, 68, 80 and 92. All routine lab assessments including labeling will be performed in the local laboratories according to the local standard and local range. An additional 100 ml blood per assessment will be drawn for the translational program in weeks 9, 26, 50, and 98 in order to evaluate the immune responses (cellular/humoral) to the vaccine/placebo. A total blood volume of 500 ml will be drawn for the translational program. Analytical test, parameters, labeling, shipment and technique of analyses for blood samples used for the translational program will be described in the separate translational program.

#### Imaging

CT or MRI imaging of the chest and abdomen alternating with US imaging of the liver for recurrence will be performed every 6 weeks in years 1 and 2 starting after the end of primary treatment phase, e.g. US after 8 weeks, CT after 14 weeks, US after 20 weeks. In year 3, CT or MRI imaging and alternating US imaging for relapse will be performed every 12 weeks. At study treatment termination CT/MRI and US imaging will be performed. CT/MRI will be performed according to local standard. Images will be evaluated according to Response Evaluation Criteria in Solid Tumors (RECIST 1.1) guidelines in order to guarantee uniformity. A maximum of 12 CT/MRI scans will be performed: at the pretreatment evaluations, following CT scans at weeks 14, 26, 38, 50, 62, 74, 86, 98, 122 and 146 and at the end of treatment evaluation (12 weeks after last treatment). Subjects who stop treatment prior to documentation of relapses will be required to undergo appropriate CT/MRI evaluation for detection of relapse. Subjects who withdraw from the study for clinical or symptomatic deterioration before objective documentation of relapse will be required to undergo appropriate CT/MRI to confirm relapse. Every reasonable effort will be made to ensure recurrence is confirmed.

## Discussion

The recurrence risk after curatively intended resection of hepatic metastases in CRC patients ranges up to 70% [15]. No generally accepted adjuvant or perioperative strategies are available, significantly reducing the recurrence rate or prolonging overall survival [9]. According to the German S3 guideline, adjuvant chemotherapy after R0 resection of liver metastases can be considered, whereas neo-adjuvant chemotherapy prior to resection of liver metastases can be considered in reasonable exceptional cases [9]. In contrast, R0 resectable metastases limited to the liver should be resected. In summary, the recommendation for neoadjuvant/adjuvant chemotherapy is currently not strong in Germany. As the majority of centers is located in Germany we decided to comply with the German guidelines, knowing that peri-operative chemotherapy is considered standard of care in some other countries.

Participating centers confirmed the acceptance of the protocol containing a placebo arm. The standard treatment of care after resection of liver metastases in the participating centers is to watch and wait. As a consequnce, the study offers a potentially effective verum treatment to two thirds of participating patients, which would not receive any treatment otherwise.

In addition, we accept patients treated in a neoadjuvant manner, if the neoadjuvant therapy has lasted 12 weeks or less. This is necessary, as some patients have been treated in a neoadjuvant setting, but the patients are presented for the study only post surgery. As a short chemotherapy does not interfere with the postoperative immune system capacity, we agreed to include those patients. A significant impact of neoadjuvant chemotherapy has not been proven so far. Thus, the ethics committee and the Paul-Ehrlich-Institute accepted inclusion criteria.

CRC is reported to have MUC1 expression in ~61% of pT1, 78% of pT2, 98% of pT3 and 90% of pT4 colorectal cancer samples and thus clearly correlates with invasiveness, being an optimal target for MUC1 based vaccination strategies after resection of hepatic metastases [34]. In addition, MUC1 expression was found in 100% (56/56) of colorectal cancer liver metastases [35]. A MUC1 expression of > 85% is expected in the study population analyzed, as the vast majority of metastatic cases will be pT3/pT4. It has been well discussed, that an engagement of the immune system might be the most successful therapeutic option to reduce micro-metastases and thus decreased recurrence rates and increase recurrence free survival time and overall survival time [36,37].

L-BLP25 is designed to induce cellular cytotoxicity towards MUC1 expressing tumor cells. Doses of 1,000 μg have increased overall survival time in NSCLC patients from 13.3 months to 30.0 months in patients with stage IIIB disease without pleural effusion (HR 0.55) [38,39]. A similar effect would be highly beneficial for patients after hepatic metastasectomy who have a high recurrence risk (70%) with a median time to recurrence of ~18-23 months [15,21].

Thus, we aim to test L-BLP-25 vaccine in a randomized, placebo-controlled, multicenter, double blinded, safety/efficacy study in patients with metastatic CRC. Eligible patients will have had their primary tumor resected and will have undergone curative-intent resection of liver metastases (R0/R1) within the last 6 weeks. Since the percentage of colorectal cancers expressing MUC1 is very high, MUC1 testing results are not required prior to inclusion. Further, the significance of detection of MUC1 expression in a tumor sample is unclear, due to the fact that this may not mean that the tumor is truly negative for MUC1, patients whose tumors are not shown to be expressing MUC1 can be included into this study. Eligible patients will be randomized via IVRS to treatment with L-BLP-25 versus placebo (2:1). Treatment will be discontinued upon documented relapse or if subjects are free from relapse, treatment will be discontinued 3 years after first application.

The dose regimen and treatment schedule will be the same as those used in Trial EMR 63325-001 (worldwide phase III, START). Throughout the development of L-BLP25, a single low-dose of cyclophosphamide (300 mg/m^2 ^to a maximum of 600 mg) has been administered prior to initiation of treatment with L-BLP25. The purpose is to overcome the immune suppression seen in cancer subjects and thus to enhance the development of an effective immune response to the MUC1 immunogen present in the vaccine.

We also decided to accept patients treated in a neo-adjuvant manner, if the neoadjuvant therapy has lasted 12 weeks or less. This became necessary, as some patients have been treated in a neo-adjuvant setting, but are presented for the study evaluation only post surgery. As a short chemotherapy seems not interfere with the postoperative immune system capacity, we agreed to include those patients in order to optimize accrual.

Patients in the investigational arm receive cyclophosphamide prior to immunotherapy, in an attempt to overcome the immune suppression seen in cancer subjects. Therefore, to maintain the double-blind design, subjects in the control arm will receive saline solution in the same calculated dose volume as that of cyclophosphamide.

The design and implementation of the current vaccination study in colorectal cancer is feasible. The study will provide recurrence-free and overall survival rates of groups in an unbiased fashion.

## Competing interests

The authors declare that they have no competing interests.

## Authors' contributions

CCS is the principal investigator. CCS, SM, and HL designed the study and participate in the performance and coordination of the trial. MS, EvC, RG, WOB, SHB, GvW, MV, MH, VH, SK, SK, FO, HS, DS, FK, VSM, IG, HL participate in the trial performance and patient recruitment. For trial management and monitoring VSM is appointed. IS the biostatistician responsible for the statistical planning. All authors have read and approved the final manuscript.

**Figure 2:**
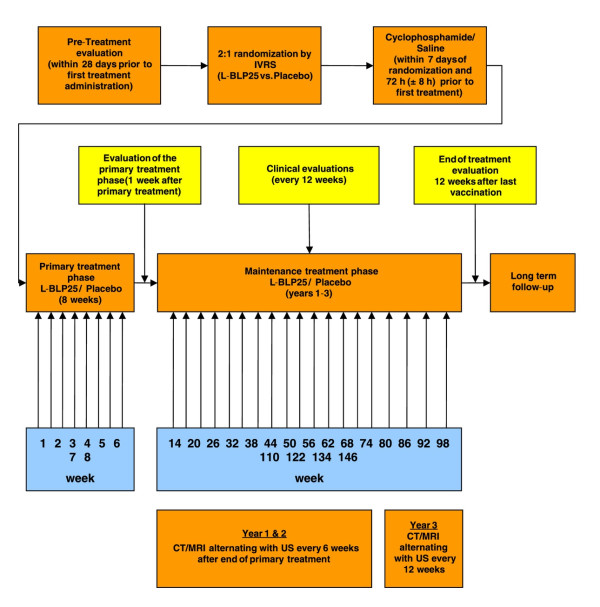
**Study design**.

## Pre-publication history

The pre-publication history for this paper can be accessed here:

http://www.biomedcentral.com/1471-2407/12/144/prepub

## Supplementary Material

Additional file 1 **Table S1 L-BLP25 Clinical Trials**. Click here for file
