# Supplementary material for: LICC: L-BLP25 in patients with colorectal carcinoma after curative resection of hepatic metastases--a randomized, placebo-controlled, multicenter, multinational, double-blinded phase II trial
Source: BMC Cancer. 2012 Apr 11;12:144. doi: 10.1186/1471-2407-12-144 (PMC3342924; doi:10.1186/1471-2407-12-144)
Supplement: Additional file 1 — Table S1 L-BLP25 Clinical Trials. [file 1471-2407-12-144-S1.DOC]

**Supplementary Table**

**Supplementary Table 1** L‑BLP25 Clinical Trials

| Protocol number Population Start date Status | Description | Subjects | | Treatment Schedule for L‑BLP25 Vaccinations^a^ | | | |
| --- | --- | --- | --- | --- | --- | --- | --- |
|  |  | Enrolled | Treated with L‑BLP25 | Primary Treatment | | Maintenance Treatment | |
|  |  |  |  | Dose | Weeks | Dose | Interval^b^ |
| NSCLC Trials | | | | | | | |
| **EMR 63325-002** Stage IIIB or IV NSCLC 04 Aug 1998 Trial closed 17 Nov 2005 | Phase I open-label safety & dose-comparison trial | 17 | 16 | 20 or  200 μg | 0,2,5,9 | 20 or  200 μg | Every 12 weeks |
| **EMR 63325-003**  Stage IIIB or IV NSCLC 06 Aug 1999 Trial closed 10 Dec 2004 | Phase II open-label safety & immunogenicity trial | 9 | 8 | 1000 μg | Weekly x 8 | 250 μg | Every 6 weeks |
| **EMR 63325-004** Stage IIIB or IV NSCLC 24 Jan 2000 Trial closed 12 Dec 2005 | Phase II open-label dose-escalation trial to determine safety & immunogenicity of L‑BLP25 in combination with L‑IL‑2 | 18 | 18 | 1000 μg combined with  5 x 10^5^ or  2 x 10^6^ IU L‑IL‑2 | Weekly x 8 | 250 μg | Every 6 weeks |
| **EMR 63325-005** Stage IIIB or IV NSCLC 08 Aug 2000 Closed to enrollment | Phase IIb open-label randomized trial to test safety & efficacy of L‑BLP25 plus best supportive care (BSC) compared to BSC alone | 171 | 88 | 930 μg | Weekly x 8 | 930 μg | Every 6 weeks |
| **EMR 63325-006** Unresected stage III NSCLC 18 Apr 2005 Closed to enrollment | Phase II open-label trial to assess safety of L‑BLP25 made with immunoadjuvant MPL® from GSK Biologicals North America | 22 | 22 | 930 μg | Weekly x 8 | 930 μg | Every 6 weeks |
| **EMR 63325-001**  Unresectable stage III NSCLC 22 Feb 2007 Open to enrollment | “START” trial. Phase III randomized, double-blind, placebo-controlled trial to test safety & efficacy of L‑BLP25 plus BSC compared to BSC alone | 1273^c^ | Blinded | 930 μg | Weekly x 8 | 930 μg | Every 6 weeks |
| **EMR 63325-009**  Unresectable stage III NSCLC 12 Feb 2009 (Step 1) | Combined phase I/II trial of L‑BLP25 in Japanese subjects with stage III unresectable NSCLC following primary chemo-radio-therapy | 7 | 6 | 930 μg | Weekly x 8 | 930 μg | Every 6 weeks |
| **EMR 63325-012** (“INSPIRE”) Unresected stage III NSCLC 02 Dec 2009  Open to enrolment | Phase III randomized, double-blind, placebo-controlled trial to test safety &efficacy of L‑BLP25 plus BSC compared to Placebo plus BSC in Asian patients | 8^c^ | Blinded | 930 μg | Weekly x 8 | 930 μg | Every 6 weeks |
| Other Trials | | | | | | | |
| **EMR 63325-007** Prostate cancer 26 Oct 2001 Trial closed 07 Jan 2005 | Phase II open-label trial to test safety & efficacy of L‑BLP25 in subjects with rising prostate-specific antigen values following radical prostatectomy | 16 | 16 | 1000 μg | Weekly x 8 | 1000 μg | Every 6 weeks |
| **EMR 63325-008**  Multiple myeloma 21 Jan 2008 Open to enrollment | Phase II randomized, open-label trial to test safety & efficacy of L‑BLP25 in combination with one or more administrations of cyclophosphamide^b^ | 34^c^ | 34^c^ | 930 μg | Weekly x 8 | 930 μg | Every 6 weeks |
| **EMR 200038-010 (“STRIDE”)** Breast Cancer  20 Oct 2009 Trial terminated July 2010 | Phase III randomized double-blind, placebo-controlled trial to test L‑BLP25 in combination with hormonal treatment versus hormonal treatment alone for first-line therapy of post-menopausal women with estrogen receptor (ER)-positive and/or progesterone receptor (PgR)-positive, inoperable locally advanced, recurrent, or metastatic breast cancer | 16^c^ | Blinded | 930 μg | Weekly x 8 | 930 μg | Every 6 weeks |
